# Uma Situação de “Apertar o Coração”: Manejando Insuficiência Cardíaca com Fração de Ejeção Reduzida em Insuficiência Adrenal Primária

**DOI:** 10.36660/abc.20230171

**Published:** 2023-07-19

**Authors:** Matheo Augusto Morandi Stumpf, Madson Queiroz Almeida

**Affiliations:** 1 Hospital das Clínicas Faculdade de Medicina Universidade de São Paulo São Paulo SP Brasil Unidade de Adrenal, Laboratório de Endocrinologia Molecular e Celular LIM/25, Disciplina de Endocrinologia e Metabologia, Hospital das Clínicas, Faculdade de Medicina da Universidade de São Paulo, São Paulo, SP – Brasil; 2 ICESP Faculdade de Medicina Universidade de São Paulo São Paulo SP Brasil Divisão de Oncologia Endócrina, Instituto do Câncer do Estado de São Paulo (ICESP), Faculdade de Medicina da Universidade de São Paulo, São Paulo, SP – Brasil

**Keywords:** Insuficiência Adrenal, Insuficiência Cardíaca, Sistema Renina-Angiotensina, Sódio

A insuficiência adrenal primária (IAP) é um fator de risco bem estabelecido para doença cardíaca isquêmica, que é a causa mais comum de insuficiência cardíaca. O tratamento de pacientes com insuficiência cardíaca com fração de ejeção reduzida (ICFEr) e IAP é discutível. Aqui, discutimos brevemente as evidências para o tratamento de ambas as condições.

Estudos nesta população em particular são escassos na literatura. Em 1983, um único estudo prospectivo apresentou um acompanhamento de longo prazo de 22 pacientes com IAP.^1^ Sete deles desenvolveram IC durante um acompanhamento médio de 30 anos. Dois deles não receberam reposição de mineralocorticoides, enquanto os cinco restantes tiveram a dose de fludrocortisona reduzida (até o máximo de 0,1 mg/dia). Três pacientes foram orientados a limitar a ingestão de sódio devido à IC grave. A IC foi predominantemente tratada com digoxina e furosemida, o tratamento habitual à época do estudo.

Recentemente, uma terapia médica direcionada por diretrizes (GDMT) para o tratamento de ICFEr incluiu betabloqueadores, inibidores do cotransportador de sódio-glicose-2 (SGLT2i), inibidores da enzima conversora de angiotensina (IECA) ou bloqueadores dos receptores de angiotensina II (BRA) ou, preferencialmente, inibidor da neprilisina e do receptor de angiotensina (ARNi), antagonista do receptor mineralocorticoide (MRA), todos associados a uma redução no risco de hospitalização e/ou taxa de mortalidade.^2^ Diuréticos são adicionados para o controle sintomático.

Os betabloqueadores podem ser usados com segurança em casos de IAP. Eles atuam inibindo a atividade simpática, prevenindo a elevação das catecolaminas, reduzindo a frequência cardíaca e diminuindo os efeitos pró-apoptóticos e cardiotóxicos na IC.^3^

Existem diversas hipóteses sobre como os SGLT2i atuam no coração. A principal teoria é o equilíbrio hídrico, que favorece a natriurese e a diurese osmótica. Outras duas possibilidades são o efeito antifibrótico direto sobre os miofibroblastos cardíacos e a remodelação do colágeno.^4^ Não existem estudos que investiguem essa classe de fármacos em pacientes com IAP. No entanto, o uso de SGLT2i na Síndrome da Secreção Inapropriada do Hormônio Antidiurético revelou maior perda de água livre,^5^ o que pode funcionar como um contrapeso à tendência de hiponatremia observada na IAP e na IC.

A IAP é acompanhada de hiperatividade do sistema renina-angiotensina, resultando em níveis elevados de angiotensina II, a qual exerce efeitos vasoconstritores e indutores de fibrose. Portanto, o uso de IECA/BRA/ARNi tem sido amplamente indicado.

O principal aspecto controverso é o uso de MRA, uma vez que a IAP é caracterizada pela deficiência endógena de aldosterona. No entanto, vale ressaltar que a produção residual de aldosterona pode ocorrer em determinadas circunstâncias.^6^

O estudo RALES é o estudo pivotal do MRA no tratamento da ICFEr.^7^ Os níveis séricos de renina e aldosterona não foram medidos para propor a terapia com o MRA, e a dose de espironolactona associada a benefícios cardiovasculares foi baixa (25-50 mg por dia). De acordo com nosso entendimento sobre outras doenças endócrinas, a espironolactona em baixas doses não é capaz de bloquear completamente a ação da aldosterona. A maioria dos pacientes com hiperaldosteronismo primário requer altas doses de espironolactona (100-200 mg por dia). Além disso, a espironolactona em baixa dose é frequentemente usada para tratar o hiperandrogenismo ovariano, não sendo associada a evidências clínicas ou bioquímicas de deficiência de aldosterona (como hipotensão, hiponatremia e hipercalemia). Ao considerarmos essas evidências, o MRA pode proporcionar benefícios clínicos na ICFEr, provavelmente devido ao bloqueio do RM nos cardiomiócitos em dose menor do que a necessária para prejudicar a ação da aldosterona nos rins.

Assim, embora alguns autores argumentem contra o uso de fludrocortisona e outros contra o uso de MRA, propomos o uso tanto de fludrocortisona quanto de MRA em baixa dose para mimetizar uma condição fisiológica, como aquela de pacientes com ICFEr sem IAP. Se o paciente tiver IAP parcial e/ou tolerar a retirada da fludrocortisona (o que é mais provável de ocorrer quando a hidrocortisona oral é administrada, já que alguma atividade mineralocorticoide é esperada), o MRA de baixa dose ainda deve ser usado pelos motivos mencionados acima.

Além disso, alguns autores acreditam que o principal ligante endógeno no RM cardíaco é um glicocorticoide, em vez da aldosterona, devido à falta de 11β-hidroxiesteroide desidrogenase tipo 2 (enzima que converte o cortisol em cortisona inativa).^8^ Como resultado, essa teoria também corrobora o uso de espironolactona mesmo quando não há reposição de mineralocorticoides.

A insuficiência cardíaca aguda induzida por fludrocortisona também foi descrita na literatura.^9 , 10^ Em ambos os relatos de casos, a suspensão da reposição de mineralocorticoide melhorou o quadro clínico e reverteu a cardiomiopatia dilatada.

Quanto às modificações do estilo de vida, a ingestão de sódio não deve ser restringida nem estimulada, com limite diário de 2300 mg (correspondente a aproximadamente seis gramas de sal). Uma dieta rica em sódio pode aumentar a hipervolemia na IC. Um estudo randomizado controlado recente (SODIUM-HF) observou que uma dieta com baixo teor de sódio (menos de 1.500 mg/d) não reduziu o desfecho composto primário (internação hospitalar relacionada a doenças cardiovasculares, visita ao pronto-socorro relacionada a doenças cardiovasculares ou morte por todas as causas em 12 meses) em pacientes com IC.^11^

**Figura 1:**
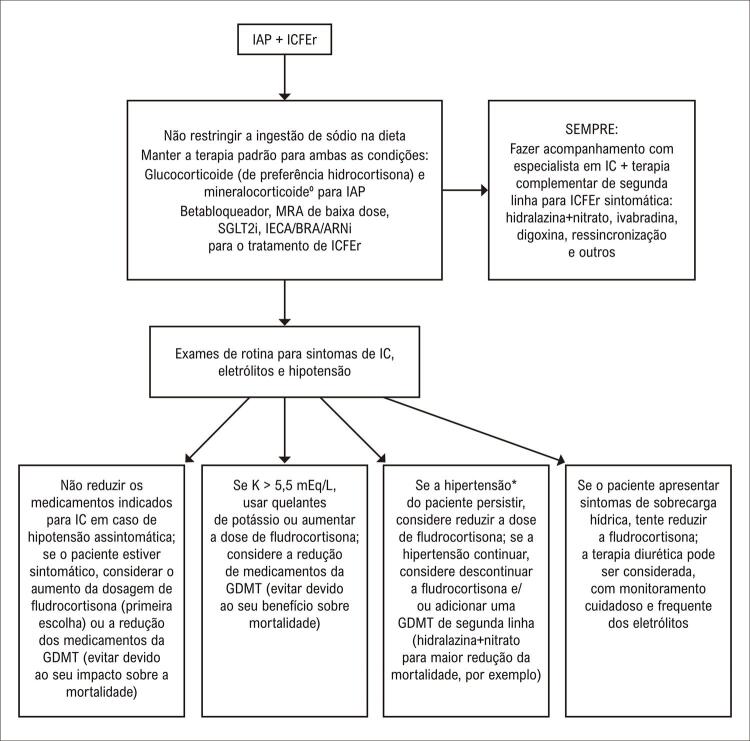
– Algoritmo sugerido para o manejo. ºNão utilizar renina ou hipotensão ortostática como parâmetro para aumento de dose; evitar mais de 0,1 mg de fludrocortisona por dia devido ao risco de agravamento da sobrecarga volêmica. *Pacientes com IAP raramente toleram as doses máximas da GDMT. ARNi: inibidor da neprilisina e do receptor de angiotensina; BRA: bloqueadores dos receptores de angiotensina; GDMT: terapia médica direcionada por diretrizes; IAP: insuficiência adrenal primária; IC: insuficiência cardíaca; ICFEr: insuficiência cardíaca com fração de ejeção reduzida; IECA: inibidores da enzima conversora de angiotensina; K: níveis séricos de potássio; MRA: antagonista do receptor mineralocorticoide; SGLT2i: inibidores do cotransportador de sódio-glicose-2.

O manejo atual da ICFEr na IAP ainda está longe de ser considerado ideal. Tratando-se de uma situação rara na prática clínica, o nível de evidência para o tratamento é baixo (a partir de pequenas séries de casos ou relatos de casos isolados). A Figura 1 apresenta uma abordagem utilizada em nossa instituição com base na opinião de especialistas e em dados coletados da população com ICFEr sem IAP.

Ensaios clínicos futuros são necessários, comparando grupos tratados com MRA com adição a fludrocortisona, MRA isolado e fludrocortisona isolada. Devido à falta de estudos de qualidade, o tratamento desses pacientes deve ser sempre individualizado. Um acompanhamento rigoroso, com reavaliação clínica e laboratorial frequente, também se faz necessário.
