# Supplementary material for: Uma Situação de “Apertar o Coração”: Manejando Insuficiência Cardíaca com Fração de Ejeção Reduzida em Insuficiência Adrenal Primária
Source: Arq Bras Cardiol. 2023 Jul 19;120(8):e20230171. [Article in Portuguese] doi: 10.36660/abc.20230171 (PMC10464856; doi:10.36660/abc.20230171)
Supplement: Translation

# Translation: A “Grasp Heart” Situation: Managing Heart Failure with Reduced Ejection Fraction in Primary Adrenal Insufficiency

**Authors:** Matheo Augusto Morandi Stumpf, Madson Queiroz Almeida

**Affiliations:** 1 Brasil Unidade de Adrenal, Laboratório de Endocrinologia Molecular e Celular LIM/25, Disciplina de Endocrinologia e Metabologia, Hospital das Clínicas, Faculdade de Medicina da Universidade de São Paulo, São Paulo, SP – Brasil; 2 Brasil Divisão de Oncologia Endócrina, Instituto do Câncer do Estado de São Paulo (ICESP), Faculdade de Medicina da Universidade de São Paulo, São Paulo, SP – Brasil

**Keywords:** Adrenal Insufficiency, Heart Failure, Renin-Angiotensin System, Sodium

Primary adrenal insufficiency (PAI) is a well-established risk factor for ischemic heart disease, the most prevalent cause of heart failure. Treatment of patients with heart failure with reduced ejection fraction (HFrEF) and PAI is debatable. Here, we briefly discuss the evidence for treating both conditions.

Trials in this particular population are lacking in the literature. In 1983, a single prospective study showed a long-term follow-up of 22 patients with PAI.^1^ Seven patients developed HF during a mean follow-up of 30 years. Two of them did not receive mineralocorticoid replacement, while the remaining five had their fludrocortisone dose reduced (to a maximum of 0.1 mg/d). Three patients were advised to limit their sodium intake due to severe HF. HF was predominantly treated with digoxin and furosemide, the usual course of treatment at the time.

Recently, a guideline-directed medical therapy (GDMT) for HFrEF included beta-blocker, sodium-glucose cotransporter-2 inhibitors (SGLT2i), angiotensin-converting enzyme inhibitors (ACEi) or angiotensin II receptor blockers (ARB) or, preferable, angiotensin receptor neprilysin inhibitors (ARNi), mineralocorticoid receptor antagonist (MRA), which are all associated with a reduction in hospitalization and/or mortality.^2^ Diuretics are added for symptom control.

Beta-blockers can be safely used in PAI. They act by inhibiting sympathetic activity, preventing catecholamine elevation, reducing heart rate, and decreasing proapoptotic and cardiotoxic effects in HF.^3^

There are several hypotheses about how SGLT2i acts in the heart. The main theory is fluid balance, which favors natriuresis and osmotic diuresis. Two additional possibilities are the direct antifibrotic effect in the cardiac myofibroblasts and collagen remodeling.^4^ There is no study of this drug class in PAI patients. However, the use of SGLT2i in the Syndrome of Inappropriate Antidiuretic Hormone Secretion revealed a greater loss of free water,^5^ which may function as a counterweight to the tendency of hyponatremia seen in PAI and HF.

PAI has a hyperactive angiotensin-renin system, resulting in high levels of angiotensin II, which has vasoconstrictor and fibrosis-induced effects. Therefore, the use of ACEi/ARB/ARNi is well indicated.

The main controversial aspect is the use of MRA since PAI is characterized by endogenous aldosterone deficiency. Nevertheless, it is important to emphasize that residual aldosterone production may occur in some circumstances.^6^

RALES study is the pivotal trial of MRA therapy in HFrEF.^7^ Serum renin and aldosterone levels were not measured to propose MRA therapy, and the dose of spironolactone associated with cardiovascular benefits was low (25-50 mg per day). According to our understanding of other endocrine diseases, low-dose spironolactone cannot completely block the action of aldosterone. Most patients with primary hyperaldosteronism require high doses of spironolactone (100-200 mg per day) for the medical treatment of aldosterone excess. Moreover, low-dose spironolactone is often used to treat ovarian hyperandrogenism and is not associated with clinical or biochemical evidence of aldosterone deficiency (such as hypotension, hyponatremia, and hyperkalemia). Considering this evidence, MRA might provide a clinical benefit in HFrEF, probably due to MR blockage in cardiomyocytes at a lower dose than would be required to impair the action of aldosterone in the kidney.

Therefore, although some authors argue against using fludrocortisone and others against using MRA, we propose using both fludrocortisone and low-dose MRA to mimic a physiological condition such as in HFrEF patients without PAI. If the patient has partial PAI and/or tolerates the withdrawal of fludrocortisone (which is more likely to occur when oral hydrocortisone is taken as there will be some mineralocorticoid activity), low-dose MRA should still be used for the reasons stated above.

Additionally, some authors believe that the main endogenous ligand in the heart MR is rather a glucocorticoid than aldosterone due to a lack of the 11β-hydroxysteroid dehydrogenase type 2 (enzyme that converts cortisol into inactive cortisone).^8^ As a result, this theory also supports the use of spironolactone even when no mineralocorticoid replacement is given.

Acute fludrocortisone-induced heart failure has also been described in the literature.^9 , 10^ In both case reports, discontinuing mineralocorticoid replacement improved the clinical condition and reverted dilated cardiomyopathy.

Concerning lifestyle modifications, sodium intake should not be restricted nor encouraged, with a daily limit of 2300 mg (corresponding to approximately six grams of salt). A high-sodium diet could enhance hypervolemia in HF. A recent randomized controlled trial (SODIUM-HF) observed that a low-sodium diet of less than 1500 mg/d did not reduce the primary composite outcome (cardiovascular-related admission to hospital, cardiovascular-related emergency department visit, or all-cause death within 12 months) in patients with HF.^11^

Management of HFrEF in PAI is still far from state of the art. Since it is a rare situation in clinical practice, the level of evidence for treatment is low (from small case series or isolated case reports). Figure 1 provides an approach used in our Institution based on expert opinion and gathered data from the HFrEF population without PAI.

**Figure 1:** – Suggested algorithm for management. ºDo not use renin or orthostatic hypotension as a parameter for dosage increases; avoid more than 0.1 mg of fludrocortisone per day due to the risk of worsening volume overload. * PAI patients rarely tolerate maximum doses of GDMT. ACEi: angiotensin-converting enzyme inhibitors; ARB: angiotensin receptor blockers; ARNi: angiotensin receptor-neprilysin inhibitor; GDMT: guideline-directed medical therapy; HF: heart failure; HFrEF: heart failure with reduced ejection fraction; K: serum potassium levels; MRA: mineralocorticoid receptor antagonist; PAI: primary adrenal insufficiency; SGLT2i: sodium-glucose cotransporter-2 inhibitors.

Future clinical trials comparing groups treated with MRA in addiction to fludrocortisone, MRA alone, and fludrocortisone alone are required. Due to the lack of high-quality studies, the treatment of these patients should always be individualized. Close follow-up, with frequent clinical and laboratory reevaluation, is also warranted.
